# Oxidative Stress in Optic Neuropathies

**DOI:** 10.3390/antiox10101538

**Published:** 2021-09-28

**Authors:** Berta Sanz-Morello, Hamid Ahmadi, Rupali Vohra, Sarkis Saruhanian, Kristine Karla Freude, Steffen Hamann, Miriam Kolko

**Affiliations:** 1 Eye Translational Research Unit, Department of Drug Design and Pharmacology, Faculty of Health and Medical Sciences, University of Copenhagen, 2100 Copenhagen, Denmark; berta.morello@sund.ku.dk (B.S.-M.); qml856@alumni.ku.dk (H.A.); rvohra@sund.ku.dk (R.V.); 2 Department of Ophthalmology, Rigshospitalet, 2600 Glostrup, Denmark; steffen.ellitsgaard.hamann@regionh.dk; 3 Group of Stem Cell Models for Studies of Neurodegenerative Diseases, Department of Veterinary and Animal Sciences, Faculty of Health and Medical Sciences, University of Copenhagen, 1870 Frederiksberg, Denmark; sarkis.saruhanian@sund.ku.dk (S.S.); kkf@sund.ku.dk (K.K.F.)

**Keywords:** oxidative stress, redox dysregulations, retinal ganglion cell, optic neuropathy, mitochondria, glaucoma, optic disc drusen

## Abstract

Increasing evidence indicates that changes in the redox system may contribute to the pathogenesis of multiple optic neuropathies. Optic neuropathies are characterized by the neurodegeneration of the inner-most retinal neurons, the retinal ganglion cells (RGCs), and their axons, which form the optic nerve. Often, optic neuropathies are asymptomatic until advanced stages, when visual impairment or blindness is unavoidable despite existing treatments. In this review, we describe systemic and, whenever possible, ocular redox dysregulations observed in patients with glaucoma, ischemic optic neuropathy, optic neuritis, hereditary optic neuropathies (i.e., Leber’s hereditary optic neuropathy and autosomal dominant optic atrophy), nutritional and toxic optic neuropathies, and optic disc drusen. We discuss aspects related to anti/oxidative stress biomarkers that need further investigation and features related to study design that should be optimized to generate more valuable and comparable results. Understanding the role of oxidative stress in optic neuropathies can serve to develop therapeutic strategies directed at the redox system to arrest the neurodegenerative processes in the retina and RGCs and ultimately prevent vision loss.

## 1. Introduction

**Figure 1:**
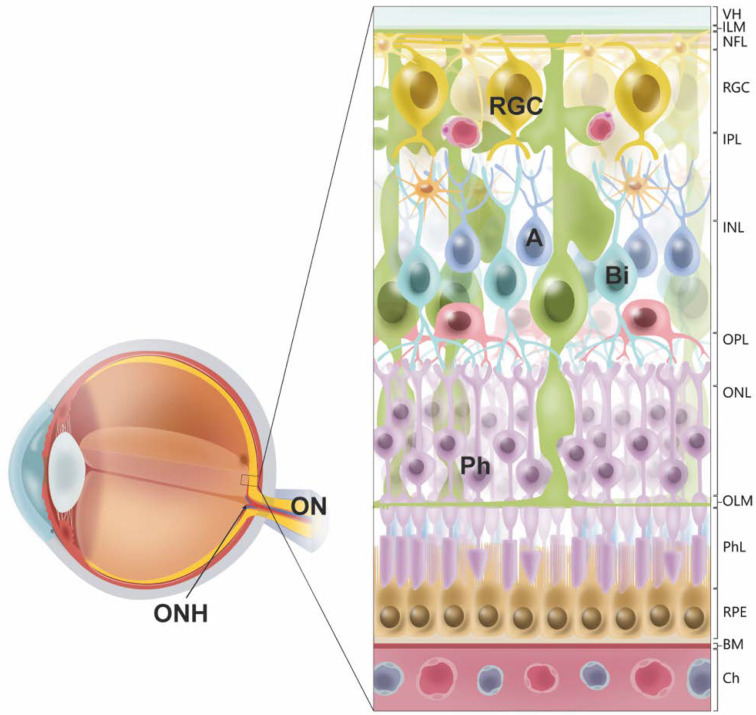
Cellular organization of the retina and location of retinal ganglion cells. Retinal ganglion cells (RGCs) localize in the innermost retinal cell layer, only separated from the vitreous humor (VH) by the inner limiting membrane (ILM). In the retina, RGCs receive inputs from amacrine cells (A) and bipolar cells (Bi), which at the same time gather visual information from the retinal photoreceptors (PR), the rods and cones. The axons of RGCs join in the nerve fiber layer (NFL) and exit the retina at the optic nerve head (ONH) to integrate the optic nerve (ON), which projects to several brain nuclei. *Abbreviations:* inner plexiform layer (IPL), inner nuclear layer (INL), outer plexiform layer (OPL), outer nuclear layer (ONL), outer limiting membrane (OLM), photoreceptor layer (PhL), retinal pigment epithelium (RPE), basal membrane (BM), choriocapillaris (Ch).

Optic neuropathies are a group of diseases characterized by the damage of the optic nerve, which usually leads to temporary or even permanent loss of vision [1,2,3,4,5,6]. The optic nerve is formed by the retinal ganglion cells (RGCs), whose axons course from the retina to the brain [7,8]. In the retina, RGCs locate in the innermost cellular layer, where they receive input from amacrine cells and bipolar cells, which simultaneously gather visual information from the retinal photoreceptors, the rods and cones [9,10] (Figure 1). RGC axons project through the nerve fiber layer on the inner surface of the retina and exit at the optic nerve head, also known as optic disc, extending up to 50 mm to several cerebral nuclei [7,8]. The most important brain nuclei for visual perception are the lateral geniculate nuclei [7,8]. RGC axons also project to the pretectal nuclei that control the pupillary light reflex; the superior colliculi, involved in visual orientation; and the suprachiasmatic nuclei that modulate the circadian rhythms [7,8]. Similar to other neurons in the mammalian central nervous system, RGCs’ regenerative capacity after injury is very limited, which affects the integrity of the visual pathway [11,12]. The etiology of most optic neuropathies is unknown. However, a growing body of evidence suggests that oxidative stress can play a role in the pathophysiology and progression of glaucoma [13,14] and might contribute to other optic neuropathies.

**Figure 2:**
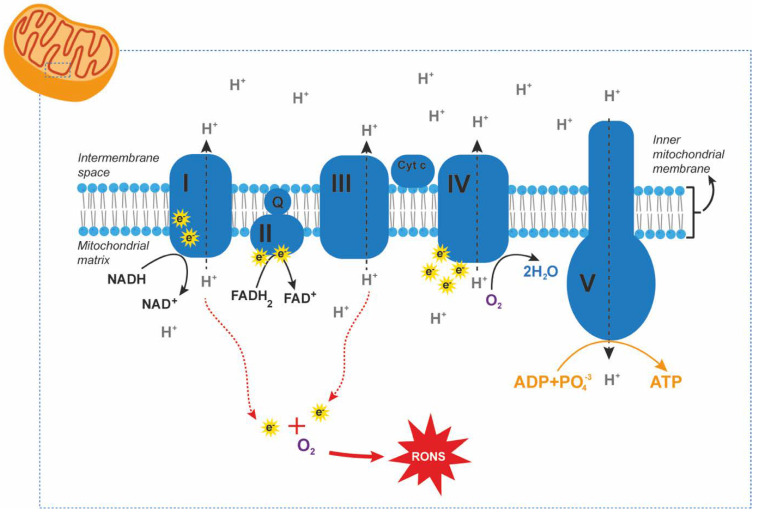
Generation of reactive oxygen and nitrogen species (RONS) by the electron transport chain. RONS are mainly generated during the last phase of cellular respiration, i.e., the electron transport chain, due to electron leakage from complex I (CI) and CIII. Mitochondria exhibit mechanisms to eliminate RONS. However, cells require the additional contribution of antioxidant enzymes like superoxide dismutase, catalase, and glutathione peroxidase to reduce cytoplasmic RONS levels and prevent cellular damage.

Oxidative stress develops when pro-oxidative processes that generate reactive oxygen and nitrogen species (RONS) overcome antioxidant defense mechanisms [15,16,17]. Energy metabolism and exposure to environmental agents are the main endogenous and exogenous sources of RONS, respectively [18]. External factors that increase RONS production are microbial infections that initiate inflammatory processes, intense physical exercise, and exposure to toxins such as cigarette smoke, alcohol, certain drugs, ionizing and UV radiations, and pollutants [18]. The main endogenous source of RONS is the electron transport chain [19,20], also known as the oxidative phosphorylation pathway, which consists of a series of protein complexes (CI-V) embedded in the mitochondrial membrane [21] (Figure 2). Electrons donated from NADH and FADH_2_ are transferred through these protein complexes; a process that is coupled to the pumping of hydrogen ions across the mitochondrial inner membrane [21]. This generates a proton gradient that is ultimately used by ATP synthase to generate ATP [21]. Oxygen acts as the terminal electron acceptor. By accepting electrons and binding to hydrogen ions, oxygen is reduced to water, a by-product of the electron transport chain [21]. However, electrons leak from CI and CIII and react with oxygen to form superoxide, which generates hydrogen peroxide, hydroxyl radical, and other RONS [19,20]. Other non-mitochondrial sources of free radicals are nitric oxide synthase reaction, Fenton’s reaction, cytochrome P450 system, peroxisomal beta-oxidation, and respiratory burst of phagocytic cells [18]. RONS are neutralized by antioxidant defense mechanisms represented by antioxidant enzymes such as superoxide dismutase (SOD), glutathione peroxidase (GPX), catalase, and their auxiliary enzymes (e.g., glutathione reductase) [22]. Low molecular weight antioxidants, either synthesized by the organism or taken with the diet, such as glutathione, ascorbic acid (vitamin C), α-tocopherol (vitamin E), and carotenoids also play an important role in buffering oxidative stress [22]. 

In this review, we will describe and summarize findings on redox dysregulations and oxidative stress in patients suffering from glaucoma, ischemic optic neuropathy, optic neuritis, hereditary optic neuropathies (i.e., Leber’s hereditary optic neuropathy and autosomal dominant optic atrophy), and nutritional and toxic optic neuropathies as well as optic disc drusen. Based on the type of samples collected and analyzed in these studies, results can be divided into systemic, where oxidative stress parameters and antioxidants in the blood, plasma, or serum samples of patients and controls (often patients with eye diseases that do not involve neurodegeneration) are investigated; or ocular analysis, where oxidative stress status and antioxidant levels are determined in aqueous humor, trabecular meshwork, or retinal tissue samples. We intend to provide an overview that can help to identify similarities in the association of oxidative stress with optic neuropathies while pointing out areas that need further investigation. This comparative study can potentially serve to develop therapeutic strategies directed at the redox system at a systemic and local level to arrest the neurodegenerative processes in the retina and optic nerve, thereby preventing vision loss.

## 2. Glaucoma

Glaucoma includes a group of diseases characterized by the progressive loss of RGCs, cupping of the optic nerve head, and associated visual field defect [1,23,24,25]. Glaucoma is broadly classified into primary and secondary glaucoma depending on the origin of the pathology. Primary glaucoma typically presents in isolation and is defined as an idiopathic disease, while secondary glaucoma is associated with predisposing conditions such as systemic diseases, clinical interventions, or trauma [1,25,26]. Glaucoma is further divided into open-angle glaucoma (OAG) and angle-closure glaucoma (ACG) [1,25]. Whereas ACG is characterized by the anatomical closure of the anterior chamber angle formed between the peripheral cornea and the eye, OAG is defined as a multifactorial disease [1,25]. Primary OAG (POAG) is the most common form of glaucoma [27]. POAG is thus more frequent than PACG worldwide (prevalence: 3.1% versus 0.5%; average number: 44.11 vs. 20.17 million), with POAG being the most prevalent in Africa (4.2%; 7.03 million) and PACG in Asia (1.1%; 15.47 million) [27], although glaucoma remains largely underdiagnosed in both continents [28]. The number of people with POAG worldwide was estimated to increase to 52.68 million by 2020 and 79.76 million by 2040; and to 23.36 and 32.04 million, respectively, in the case of PACG, which represents a major health and economic burden [27]. The main known risk factors for developing POAG are older age and elevated intraocular pressure (IOP) [26,29]. However, POAG is not always associated with high IOP [25,30]. Thus, POAG is further classified into high-tension glaucoma (HTG) and normal-tension glaucoma (NTG). NTG patients exhibit the typical pathological features of RGC degeneration but IOP within the normal range [25,30]. Moreover, despite well-adjusted IOP, 15% of glaucoma patients become blind and as many as 42% lose sight in one eye [31]. In this regard, growing evidence obtained from both animal models and clinical studies has revealed risk factors other than elevated IOP and aging, which include vascular dysfunction [32,33,34,35], glutamate excitotoxicity [36,37,38], mitochondrial dysfunction [39,40,41], and oxidative stress [13,14,42,43,44]. Given its higher prevalence, a vast proportion of studies attempting to decipher the association between oxidative stress and glaucoma have focused on POAG. The main results of these studies and others investigating oxidative stress in PACG and secondary glaucoma have been summarized in Table 1. Alterations in oxidative stress observed in other optic neuropathies different from glaucoma have been summarized in Table 2.

### 2.1. Oxidative Stress in Primary Glaucoma

#### 2.1.1. Alterations in Antioxidant Defense Mechanisms in Primary Glaucoma

Several parameters have been analyzed to determine the antioxidant potential of samples from glaucoma patients. The most cited in the literature are total antioxidant capacity (TAC), total antioxidant status (TAS), biological antioxidant potential (BAP), and total reactive antioxidant potential (TRAP) [43,45,46,47,48,53,54,63,81]. These parameters and the assays used to measure them are similar and their results largely correlate, making them comparable [82,83]. BAP was found to be lower in peripheral blood samples of POAG patients and HTG patients in a Japanese population [47]. Lower BAP levels correlated with worse visual field sensitivity [47] and a lower number of RGCs in POAG patients below 65 years of age [48]. Also, TAS has been shown to be decreased in Saudi Arabian POAG patients, and low TAS was found to correlate with increased glaucoma severity reported as a higher cup-to-disc ratio [46]. Similarly, plasma samples from Caucasian POAG patients showed lower TAC [45]. Rokicki et al. found no changes in TAC, but elevated total oxidant status (TOS) in plasma samples of POAG patients in a comparable case-control study [63]. Interestingly, patients with ocular hypertension (OHT) not suffering glaucomatous degeneration have been shown to exhibit a higher TAC than NTG patients and controls, which has been hypothesized to protect them from RGC loss and visual damage [43,81]. Ocular analysis of antioxidant capacity appears to be consistent with the results reported by systemic studies. TAC [45], TAS [53], and TRAP [54] have been shown to be lower in the aqueous humor of POAG, especially in subjects who do not receive treatment to modulate their elevated IOP [53]. 

Glutathione is the most abundant endogenous antioxidant in the body [84,85]. This thiol neutralizes lipid peroxides and reactive oxygen and nitrogen species, which leads to a change in its redox state from reduced glutathione (GSH) to oxidized glutathione (GSSG) [84,85]. In healthy cells and tissues, more than 90 percent of glutathione is in the reduced form, this being the enzyme glutathione reductase responsible for regenerating GSH from GSSG [84,85]. Higher levels of GSSG and a lower redox index (logarithmic value of the GSH-to-GSSG ratio) were observed in peripheral blood mononuclear cells of POAG patients compared to controls suffering from other eye conditions different from glaucoma (e.g., traumatic optic neuropathy) [57]. In addition, the redox index correlated with visual field damage in POAG patients [57]. However, some other studies showed that subjects with POAG exhibit lower GSH levels but unchanged GSSG levels in the blood compared to controls [58,59]. These differences could not be due to the type of sample analyzed (blood mononuclear cells versus whole blood samples) since glutathione concentration in plasma is very low (in the range of micromolar) [86]. They may rather be due to the inclusion criteria, i.e., analysis of samples from untreated POAG patients and control subjects with no previous history of any eye condition [58,59]. In addition, analysis of samples from well-characterized HTG and NTG patients allowed the identification of a lower redox index only in HTG [59]. Finally, analysis of total thiol and their oxidized form (i.e., disulfide) in serum has reported a higher disulfide-to-native thiol ratio in patients with POAG [61]. 

The specific activity and expression of important enzymes involved in antioxidant defense mechanisms such as SOD, GPX, and catalase have also been studied in patients with glaucoma. SOD converts the free radical superoxide to oxygen and hydrogen peroxide by oxidation and reduction, respectively [87]. In humans, we can find three different types of SOD (SOD1–3) [88]. SOD1 is highly abundant in the cytosol and targets superoxide produced in both the cytosol and the mitochondria, SOD2 is located exclusively within the mitochondria, and SOD3 is localized in the extracellular matrix [88]. Genetic expression of SOD1 was shown to be downregulated in blood samples from POAG patients, while SOD2 expression was upregulated [62]. Enzymatic activity analyses showed that SOD2 activity in serum was lower in POAG patients compared to patients with cataracts [63]. However, SOD1 activity was found to be unaffected [63]. On the contrary, ocular studies have shown that total SOD activity is increased in the aqueous humor of POAG patients [53,54,66,67], especially when patients are not being treated to control IOP [53]. Similar findings have been observed in PACG patients [67]. GPX represents a family of several enzymes that reduce hydrogen peroxide to water or lipid hydroperoxides to their corresponding alcohols using GSH as an electron donor [89]. In humans, four major isoforms of GPX (GPX1–4) have been found, being GPX1 the most abundant subtype [90]. Interestingly, the genetic expression of GPX1 was found to be upregulated in POAG patients [62]. Moreover, total GPX activity has been found to be increased in the aqueous humor of POAG [54,66,67] and PACG patients [67]. Finally, catalase metabolizes hydrogen peroxide to water and oxygen [91]. This enzyme is mainly localized in peroxisomes [92] and, to a lesser extent, in the cytosol [93]. Catalase activity appears to remain unchanged in the aqueous humor of POAG and PACG patients [54,66,67] and, to our knowledge, it has not been measured in blood samples from patients.

#### 2.1.2. Overproduction of Reactive Oxygen and Nitrogen Species (RONS) in Primary Glaucoma

Studies of RONS levels in glaucoma patients have focused primarily on nitric oxide (NO). NO is a free radical that carries important physiological functions such as smooth muscle relaxation, vasodilation, neurotransmission, and inflammatory regulation [94]. In the eye, NO promotes drainage of aqueous humor by relaxing the trabecular meshwork [95], and controls local blood circulation [96] together with endothelin-1 (ET-1), a vasoconstrictor released from endothelial cells [97,98]. However, uncontrolled NO production can lead to oxidative stress and apoptosis [99,100]. Aqueous humor NO levels have been shown to be elevated in POAG and PACG patients compared to patients with cataracts [70,101]. Systemic inhibition of NOS in glaucoma patients decreases blood flow in the optic nerve head (ONH) but not to the same extent as in healthy subjects [102]. This indicates that NO upregulation might be a compensatory mechanism to maintain an appropriate supply of oxygen and nutrients to the retina [102]. Furthermore, analyses of trabecular meshwork samples reported an upregulation of inducible NO synthase (iNOS) and a downregulation of calcium/calmodulin-dependent NOS expression and activity, which correlated with visual field defects in POAG patients [71]. Moreover, increased plasma and aqueous humor ET-1 levels have been observed in glaucoma patients [101,103,104,105,106]. Alterations in NO and ET-1 levels most probably affect ocular blood flow by dysregulating the balance between vasodilation and vasoconstriction, which possibly impacts RGC metabolic supply and increases oxidative stress, leading to RGC neurodegeneration [107]. Additionally, defective coupling of the ocular neurovascular unit possibly due to reactive gliosis and decreased neuronal activity has been associated with poor visual function [108,109].

In addition to NO, some clinical studies have investigated the association between ferritin, the main storage regulator of iron [110], and primary glaucoma. Free iron promotes the formation of reactive hydroxyl radicals, which induces oxidative stress and cell toxicity [111], and ferritin is commonly used as a marker of iron-related oxidative stress [111,112]. High levels of ferritin in plasma have been associated with glaucoma in two separate South Korean population studies [68,69], particularly among men [68]. Consistent with this, subjects under iron supplementation are more likely to be diagnosed with glaucoma [113].

#### 2.1.3. Oxidative Stress Markers in Primary Glaucoma

Oxidative stress affects all biomolecules, including lipids, proteins, and especially DNA, which can lead to apoptosis [18,114]. Several studies have found elevated levels of the DNA oxidative marker 8′-hydroxy-2′-deoxyguanosine (8-OHdG) in plasma [72,73,74], aqueous humor [73], and trabecular meshwork [76,77] samples of POAG patients compared to controls. Furthermore, DNA oxidative damage in the trabecular meshwork has been shown to correlate with visual field defects [77]. Deleterious DNA modifications such as alkylation, deamidation, and oxidation are repaired primarily by base excision repair (BER) mechanisms in both dividing and non-dividing cells [115]. POAG patients were found to express lower plasma levels of the BER enzymes poly (ADP-ribose) polymerase 1 (PARP1) and oxoguanine DNA glycosylase 1 (OGG1) [73]. Mohanty et al. also found a negative correlation between PARP1 and OGG1 expression and 8-OHdG plasma levels in these patients [73]. Moreover, analysis of the association between BER gene polymorphisms and POAG in a Polish population identified that the 399Arg/Gln genotype of X-ray repair cross-complementing 1 (XRCC1) gene was associated with a higher risk of POAG [116]. 

Lipid peroxidation and cellular oxidative stress are identified by the marker malondialdehyde (MDA) [117,118]. Systemic studies have reported higher levels of MDA in the blood of POAG patients [45,63,74]. However, measurements of MDA levels in the aqueous humor of POAG patients have found conflicting results: either increased MDA levels [45,79] or no changes in this lipid oxidative marker [71]. Fernandez-Durango et al. excluded POAG patients with normal IOP (<21 mm Hg) from their study, which may account for these discrepancies. Moreover, plasma concentrations of several hydroxylinoleate and hydroxyarachidonate isomers, which are oxidation products of the abundant polyunsaturated fatty acids linoleic acid and arachidonic acid, have been found to be increased in both NTG and HTG patients [78]. 

Finally, protein oxidative damage has also been observed in patients with POAG. High levels of NO interact with the superoxide anion, generating peroxynitrite. Peroxynitrite induces the nitration of several amino acids, including tyrosine, which leads to the formation of nitrotyrosine [119]. Nitrotyrosine immunoreactivity has been observed to be significantly higher in the trabecular meshwork [71] as well as in the blood vessels and glia located in the pre-laminar optic nerve head [80] of patients with POAG. Interestingly, elevated nitrotyrosine immunoreactivity in the trabecular meshwork has been shown to correlate with elevated IOP in POAG patients [71].

### 2.2. Oxidative Stress in Secondary Glaucoma

#### 2.2.1. Oxidative Stress in Pseudoexfoliation Glaucoma

Pseudoexfoliation glaucoma (PEXG) is the most common type of secondary glaucoma, especially of secondary OAG, although it has also been associated with secondary ACG [120]. PEXG is characterized by the progressive deposition of extracellular matrix proteins that form a fibrillar material in the anterior segment of the eye, most notably on the anterior lens capsule and the pupillary border, although these fibers also accumulate in the cornea, ciliary body, trabecular meshwork, and other ocular tissues [121]. Accumulation of aberrant fibrillar material on the trabecular meshwork and Schlemm’s canal impairs aqueous humor drainage, increasing IOP in 25% of patients, and correlates with glaucoma [121]. Other pathological events caused by deposition of the exfoliation material, such as progressive iris degeneration and the associated accumulation of melanin granules, as well as changes in the ocular vasculature and damage of the lamina cribosa are also thought to contribute to glaucomatous neurodegeneration [121]. PEXG is a late-onset and complex condition that typically develops faster and more aggressively than POAG [121,122]. Both genetic and environmental factors contribute to PEXG development, and oxidative stress has been postulated to play an important role in the disease pathogenesis [123].

TAS [49,50] and TAC [51,52] are lower in the plasma of PEXG patients. Similarly, TAC [51] and TRAP [55] have been found to be lower in aqueous humor samples from PEXG patients. In agreement with this, TOS in plasma [51,124] and aqueous humor samples [51] has been shown to be elevated in PEXG patients compared to controls. However, Ergan et al. have reported higher TAS and no differences in TOS in the aqueous humor of PEXG patients compared to controls [56], which might be explained by the small sample size used in this particular study.

The activity of antioxidant enzymes has also been studied in PEXG with conflicting results. Some studies have shown higher levels of total SOD activity in the plasma [124] and aqueous humor [55] of patients with PEXG. However, a recent study demonstrated decreased total SOD activity in the plasma of PEXG patients [60]. Although Yaz et al. included a relatively high sample size in their study, they did not exclude participants with systemic diseases if those were controlled with medication, which could act as a confounding factor and explain the different results of the study. For instance, insulin administration is known to modulate Nrf2 dependent antioxidant enzymes, including SOD [125]. Moreover, catalase activity has been shown to be consistently lower in both plasma/blood [60,65] and aqueous humor [65] samples of patients with PEXG. Finally, total GPX activity was found to be increased in the aqueous humor of PEXG patients [55].

The analysis of pro-oxidant and antioxidant molecules in PEXG has also led to conflicting findings. Plasma NO levels in PEXG patients have been found to be both elevated [52] and decreased [60] compared to healthy controls. By contrast, ET-1 levels have been consistently reported to be elevated in both plasma and aqueous humor samples from PEXG patients, which correlates with reduced ocular blood flow and may contribute to optic nerve damage [106,126,127,128]. One study also reported higher plasma GSH levels in PEXG patients [60], which could act as a compensatory mechanism to buffer excess of RONS. The dysregulation between oxidants and antioxidant defense mechanisms in PEXG has been further suggested due to the presence of high levels in plasma of oxidative damage markers such as MDA [52,60] and 8-OHdG [75]. Therefore, oxidative stress seems likely to be involved in PEXG pathogenesis. To further support this hypothesis, new studies involving a higher number of patients and controls, more strict selection criteria (e.g., exclusion of subjects with systemic diseases other than exfoliation syndrome), and analysis of a wider range of pro-oxidant and antioxidant agents, particularly in the eyes of PEXG patients, would be necessary.

#### 2.2.2. Oxidative Stress Associated with Ocular Surgery

Ocular surgical interventions such as vitrectomy have been suggested to contribute to the development of secondary OAG [129,130]. The main hypothesis to explain this causal relationship is that vitrectomy increases the exposure of the eye microenvironment to oxygen, disturbing the oxidant–antioxidant equilibrium [131]. A study showed that patients who had previously undergone vitrectomy exhibited increased oxygen levels in the anterior chamber, the anterior chamber angle, the lens, and the posterior chamber compared to patients with no history of eye interventions or POAG that were scheduled for cataract surgery [132]. Furthermore, it was found that the ocular levels of TRAP and ascorbic acid, another important antioxidant found in mammalian aqueous humor [133], were lower in patients after vitrectomy [132]. Siegfried & Shui (2019) suggested that increased oxygen exposure after vitrectomy would increase RONS in the anterior and posterior eye chambers and damage the trabecular meshwork. This would reduce aqueous humor drainage, increase IOP, and lead to glaucoma [132]. Further evidence such as oxidative damage in trabecular meshwork samples or altered expression and activity of antioxidant enzymes is required to support this proposed pathological mechanism.

**Table 2:** Other optic neuropathies different from glaucoma.

Type of Condition	Type of Sample	Outcome (Related to Control Group) *	Country	Authors
General antioxidant/oxidant status				
NAION	Plasma	No changes in TAS	Turkey	[134]
NAION	Plasma	No changes in TOS	Turkey	[134]
Optic neuritis	Blood	Positive correlation between the disulfide-to-native thiol ratio and P100 wave latency	Turkey	[135]
LHON (m.11778G>A, m.14484T>C, or m.3460G>A)	Plasma	Lower TAS	Serbia	[136]
LHON (m.11778G>A, m.14484T>C, or m.3460G>A)	Plasma	Higher TOS (only when comparing female LHON carriers against female controls)	Serbia	[136]
LHON (m.11778G>A, m.14484T>C, or m.3460G>A)	Plasma	Higher OSI	Serbia	[136]
LHON (m.11778G>A)	Peripheral blood cells	Higher cell death after incubation with 2-deoxy-d-ribose	Italy	[137]
ADOA	Lymphocytes	Increased susceptibility to oxidative stress and cell death after incubation with 2-deoxy-d-ribose	Italy	[138]
ADOA	Fibroblasts	Positive correlation between mitochondrial calcium uptake and cell death and symptom severity	Hungary	[139]
Antioxidant defense mechanisms				
Optic neuritis	Serum	Lower bilirubin levels	China	[140]
NMO	Serum	Lower bilirubin levels	USA	[141]
LHON (m.11778G>A)	Blood	Lower levels of α-tocopherol (vitamin E)	Hungary	[142]
LHON (m.11778G>A, m.14484T>C, or m.3460G>A)	Fibroblasts	Unaffected LHON carriers show higher expression of transcription factors and enzymes related to antioxidant pathways (compared to affected LHON carriers) *	Italy/Brazil	[143]
Toxic optic neuropathy (ethambutol)	Blood	Lower levels of SOD and catalase (especially in diabetic patients)	Saudi Arabia	[144]
Nutritional optic neuropathy	Blood	Decreased folate concentrations in subjects with optic neuropathy	Papua New Guinea	[145]
Nutritional optic neuropathy	Serum	Higher concentrations of thiamine (B12), riboflavin (B2), niacin (B3), and lycopene linked to a decreased risk of developing optic neuropathy	Cuba	[146]
Reactive oxygen and nitrogen species (RONS) and pro-oxidative enzymes				
NMO	Serum	Higher GGT levels	China	[147]
LHON (m.15927G>A)	Cybrid cell lines	Higher production of RONS	China	[148]
ADOA	Fibroblasts	Normal production of RONS	Italy	[149]
Oxidative stress markers				
NAION	Plasma	No changes in AOPP levels	Turkey	[134]
LHON (m.11778G>A, m.14484T>C, or m.3460G>A)	Plasma	Higher AOPP levels	Serbia	[136]
LHON (m.11778G>A, m.14484T>C, or m.3460G>A)	Fibroblast proteins	Increased S-glutathionylation	Singapore	[150]
LHON (m.11778G>A)	Leukocytes	Higher 8-OHdG levels	Taiwan	[151]
Genetic alterations related to oxidative stress				
NAION	Blood	Higher prevalence of loss-of-function deletion genotype in GSTM1	Saudi Arabia	[152]
NAION	Blood	Higher prevalence of loss-of-function deletion genotype in GSTM1	China	[153]
NAION	Blood	Higher levels of non-synonymous mutations in mtDNA and a higher content of relative mtDNA	USA	[154]
NAION	Leucocytes	Negative correlation between mtDNA relative content and visual acuity	Saudi Arabia	[155]
Optic neuritis	Blood	Higher prevalence of loss-of-function deletion genotype in GSTT1	Saudi Arabia	[152]
ADOA	Fibroblasts	Increased depletion of mtDNA	UK	[156]

Abbreviations: ADOA: autosomal dominant optic neuropathy, AOPP: advanced oxidation protein products, GGT: gamma-glutamyltransferase, GSTM1: glutathione s-transferase Mu 1, GSTT1: isoform of glutathione s-transferase theta 1, LHON: Leber’s hereditary optic neuropathy, mtDNA: mitochondrial DNA, NAION: non-arteritic ischemic optic neuropathy, NMO: neuromyelitis optica, OSI: oxidative stress index, TAS: total antioxidant status, TOS: total oxidant status, SOD: superoxide dismutase, 8-OHdG: 8′-hydroxy-2′-deoxyguanosine. * Control group: may refer to patients with other eye diseases not associated with optic nerve degeneration (e.g., cataracts), healthy unrelated subjects (e.g., spouses), healthy relatives (e.g., mutation carrier not suffering the optic neuropathy), etc.

## 3. Ischemic Optic Neuropathy

Ischemic optic neuropathy (ION) is the most common acute optic neuropathy in patients over 50 years of age, and it refers to a group of conditions in which the integrity and function of the optic nerve are affected due to a vascular insufficiency [157,158,159,160,161]. ION presents most often as a sudden, painless, unilateral visual acuity loss event associated with optic disc edema and a significant reduction in the visual field [161]. ION can be divided into non-arteritic ION (NAION) and arteritic ION (AION), where the latter is most often caused by giant-cell arteritis [2]. The exact cause of NAION remains unknown, although it has mainly been associated with anatomical risk factors such as a small optic cup-to-disc ratio [162,163,164] and optic disc drusen (ODD) [165,166], as well as vascular risk factors like hypertension and diabetes mellitus, present in 50% and 25% of patients, respectively [2]. Other vascular risk factors that lead to ocular perfusion alterations and/or ischemia include hypercholesterolemia, atherosclerosis, obstructive sleep apnea, and stroke [157,158,160,161]. Since oxidative stress has been linked to other optic neuropathies and is known to affect the microvasculature, leading to vasoconstriction and vascular remodeling that affect microcirculation [167], some studies have begun to explore the possible implication of oxidative stress in NAION (Table 2). 

### Oxidative Stress in Non-Arteritic Ischemic Optic Neuropathy

Two separate studies in Saudi Arabia [152] and China [153] have shown that the prevalence of a loss-of-function deletion genotype in *GSTM1* was significantly higher among NAION patients compared to controls. *GSTM1* encodes one of the isoforms of glutathione s-transferase, an important enzyme involved in the detoxification of oxidative stress products, among other toxins, by conjugation with glutathione [168]. Moreover, NAION patients have been found to exhibit higher levels of non-synonymous mutations in mitochondrial DNA (mtDNA) [154] and a higher content of relative mtDNA [155]. Relative mtDNA content is known to increase in response to impairments in oxidative phosphorylation and reduced ATP synthesis, which typically occurs with age and oxidative stress [169,170]. Interestingly, relative mtDNA content has been shown to correlate negatively with visual acuity [155]. Therefore, oxidative stress and mitochondrial alterations may be risk factors for NAION. However, the first systemic study analyzing plasma samples from NAION patients did not show differences in the levels of TAS, TOS, and advanced oxidation protein products (AOPP) when compared to those of healthy controls [134]. Ocular blood flow fluctuations and ischemia and/or genetic alterations associated with the redox system may increase RONS levels at the ONH and retina, contributing to RGC degeneration [171]. Further research is needed to explore this hypothesis to determine the implications of oxidative stress in vascular alterations associated with NAION. 

## 4. Optic Neuritis

Optic neuritis is the most common acute optic neuropathy among patients under 50 years of age [172,173,174]. It is defined as an acute inflammatory condition that affects the optic nerve and can cause temporary or permanent vision loss depending on the etiology, time of diagnosis, and appropriate treatment [3]. Optic neuritis can present as a single or repeated episode(s) and can be isolated to the optic nerve or associated with other disorders of the central nervous system (CNS) such as multiple sclerosis (MS) or neuromyelitis optica (NMO) [175,176]. Additional risk factors for this optic neuropathy are other autoimmune disorders, infections, granulomatous disease, and processes that induce demyelination, including oxidative stress [3]. The implications of oxidative stress in MS have been thoroughly reviewed elsewhere [177,178,179]. Therefore, we will mainly summarize alterations in antioxidant and pro-oxidants in optic neuritis and NMO (Table 2). 

### Oxidative Stress in Optic Neuritis and NMO

A recent study assessed the thiol-disulfide homeostasis, an indicator of redox imbalance, in blood samples from patients with MS experiencing an episode of optic neuritis [135]. The results revealed a positive correlation between the delay in nervous transmission from the retina to the brain, known as the P100 wave latency, and the disulfide-to-native thiol ratio, i.e., the relative proportion of oxidized thiol against the entire thiol pool [135]. Furthermore, the levels of serum gamma-glutamyltransferase (GGT), a ubiquitous enzyme with a pro-oxidant role [180,181], are higher in NMO patients compared to healthy controls, patients with MS, and patients with Parkinson’s disease, i.e., a non-inflammatory neurological disease [147]. Bilirubin, which for a long time was only considered as a waste product of heme catabolism, is an important endogenous antioxidant comparable to ascorbic acid and catalase [182]. Importantly, serum bilirubin levels are decreased in patients with optic neuritis compared to healthy controls and as low as those observed in NMO patients [140]. A separate study found similar results when comparing serum bilirubin levels in NMO patients and healthy subjects [141]. Moreover, Abu-Amero et al. showed that patients with optic neuritis often carry a deletion in *GSTT1*, a gene that encodes another isoform of glutathione s-transferase, which reduces its enzymatic activity [152]. More recently, imaging studies have identified reductions in retinal blood vessel density and perfusion as well as decreased thickness of the retinal nerve fiber layer (RNFL) in patients with optic neuritis [183,184,185]. Also, a lower number of perivascular astrocytes has been observed in optic neuritis and NMO lesions, suggesting alterations in the neurovascular unit coupling [186,187]. Evidence of whether such vascular and glial changes are associated with increased oxidative stress and lead to RGC loss needs to be presented. In addition, further cohort studies and a more detailed evaluation of the expression and activity of antioxidant enzymes (e.g., GPX) as well as oxidative markers (e.g., 8-OHdG) are needed to determine the role of redox dysregulations in optic neuritis.

## 5. Hereditary Optic Neuropathies

### 5.1. Leber’s Hereditary Optic Neuropathy (LHON)

Leber’s hereditary optic neuropathy (LHON) is a major cause of blindness associated with mitochondrial dysfunction and typically seen in young adults (~30 years old) [188,189]. LHON presents with painless, subacute visual loss in one eye, followed by a subsequent visual loss in the fellow eye within a time interval of 3–6 months [4,190]. However, in around 25% of cases, both eyes are affected simultaneously [191]. The prevalence of LHON is ~1 in 30.000 and it represents the most common primary mitochondrial DNA (mtDNA) disease [188,189,192]. Approximately 90% of patients with LHON carry one of three mtDNA point mutations affecting Complex I subunits in the electron transport chain: m.3460G>A, m.11778G>A, and m.14484T>C [193,194]. The m.11778G>A mutation is the most common cause of LHON worldwide as it accounts for 70–90% of all cases. In addition, LHON is characterized by a low penetrance and a higher incidence among male carriers (50% vs. 10% in female carriers) [4]. In this regard, LHON is considered a complex multifactorial disease triggered by environmental agents, particularly smoking [195], and possibly influenced by hormonal factors given the gender bias observed among LHON carriers [196]. RGC loss in LHON is thought to be due to a combination of decreased adenosine triphosphate (ATP) synthesis and increased generation of RONS levels due to impaired mitochondrial oxidative phosphorylation (Table 2).

#### Oxidative Stress in LHON 

A recent study analyzing plasma samples from patients with LHON showed several alterations related to oxidative stress that were consistent among LHON patients who had different mitochondrial mutations (m.11778G>A, m.14484T>C, or m.3460G>A) [136]. In particular, plasma of LHON patients showed increased TOS (only when comparing female LHON carriers against female controls), decreased TAS, and higher oxidative stress index (OSI), indicating an imbalance in the redox status [136]. This is consistent with a few preliminary studies reporting lower circulating levels of α-tocopherol (vitamin E) [142] and higher cell death of peripheral blood cells incubated with 2-deoxy-d-ribose, a sugar that induces oxidative stress and apoptosis by depleting GSH content, in LHON patients (m.11778G>A) [137]. Interestingly, Rovcanin et al. found that both symptomatic and asymptomatic LHON patients showed similar oxidative stress alterations, suggesting that mtDNA point mutations per se induce a pro-oxidative phenotype [136]. Also, some studies indicate that LHON might be a neurovascular disease since decreased vascular density in the retina is observed in both unaffected and affected LHON carriers and precedes RNFL thinning [197,198,199,200,201]. Furthermore, smoking has been shown to negatively influence TAS and OSI in LHON patients [136].

A study that directly analyzed the effect of cigarette smoke condensate (CSC) on fibroblasts from affected LHON patients and unaffected LHON carriers (m.11778G>A, m.14484T>C, or m.3460G>A) found that CSC reduces mtDNA copy number and ATP synthesis in both cell lines similar to what is observed in control cells [143]. However, fibroblasts from unaffected LHON patients showed a strong compensatory mechanism after CSC exposure determined by the expression of mitochondrial biogenesis markers [143]. Additionally, under normal conditions, fibroblasts from unaffected LHON carriers were shown to express higher levels of transcription factors and enzymes involved in antioxidant pathways than those from affected LHON patients [143]. Following treatment with CSC, protein levels of antioxidant factors increased equally in fibroblasts from controls, unaffected, and affected LHON patients [143]. Moreover, a study analyzing human induced pluripotent stem cells (hiPSCs)-derived RGCs of affected and unaffected LHON patients carrying the most frequent mtDNA point mutation (i.e., m.11778G>A) found alterations in the transport of mitochondria [202]. Both LHON cell lines showed increased RONS production. However, only hiPSCs-derived RGCs of affected LHON patients exhibited an increased number of retrograde mitochondria and reduced number of stationary mitochondria in the RGC axons as well as increased apoptosis [202]. In line with this, the expression of KIF5A, a kinesin involved in microtubule transport, was found to be significantly reduced in RGCs of affected LHON patients [202]. 

In agreement with these changes, markers of oxidative stress damage have been consistently identified in LHON. For instance, higher levels of 8-OHdG have been detected in leukocytes from LHON patients (m.11778G>A) and asymptomatic maternal relatives [151]. The plasma of LHON patients (m.11778G>A, m.14484T>C, or m.3460G>A) also showed higher levels of AOPP [136]. Furthermore, fibroblast proteins in LHON patients (m.11778G>A, m.14484T>C, or m.3460G>A) exhibit increased S-glutathionylation [150], which refers to the reversible binding of glutathione to cysteine residues in target proteins as a consequence of direct oxidation or due to thiol–disulfide exchange, leading to loss of function [203]. This protein modification was shown to affect proteins related to energy metabolism, catalytic activity, and cell protein quality control [150]. Importantly, inhibition of Complex I in fibroblasts from healthy subjects led to a similar pattern of S-glutathionylation, indicating that Complex I misfunction is primarily responsible for this oxidative stress-related molecular process [150]. 

Although the implication of Complex I dysfunction in LHON is obvious, phenotypic differences between LHON patients point out the possibility that other molecular factors are involved in this optic neuropathy. This was demonstrated in a Chinese family carrying m.11778G>A, which exhibited a high penetrance of LHON [204]. Lymphoblastoid cell lines derived from five subjects of this family showed decreased activity of both Complex I and III, causing a more pronounced reduction in ATP synthesis and increase in RONS levels than that observed in mutant cell lines from other families [204]. Furthermore, another recent study that screened 352 Han Chinese subjects with LHON lacking the known mtDNA mutations found that eight subjects carried the tRNAThr 15927G>A mutation [148]. Such mutation was found to interfere with the translation of mitochondrial proteins and was associated with a decreased activity of Complex I and III, lower ATP synthesis, and higher RONS production when compared to control subjects [148]. Further research is needed to determine the implication of other nuclear and mitochondrial elements that might be responsible for the different penetrance of LHON as well as their presence in different ethnic groups. 

### 5.2. Autosomal Dominant Optic Atrophy (ADOA)

Autosomal dominant optic atrophy (ADOA) is a dominantly inherited optic neuropathy that typically develops in the second decade of life, although the age of onset varies greatly [205,206]. It affects ~1 in 12,000 to 35,000 subjects and is thereby the most common inherited optic neuropathy [207,208]. The visual loss progresses slowly and ranges from mild visual alterations to severe blindness [207,209]. ADOA is usually caused by mutations in *OPA1* (60-80% of the cases), but it has been also associated with mutations in *OPA3*, *OPA4*, *OPA5,* and *OPA8* [210]. *OPA1* encodes a mitochondrial dynamin-like GTPase protein that helps to control cristae remodeling, maintenance of mitochondria membrane integrity, and mitochondria fusion, thereby regulating mitochondrial shape and function [211,212] and mitochondrial genome maintenance [213]. Therefore, mutations in *OPA1* inevitably result in a decline of mitochondrial function and subsequent ATP production by oxidative phosphorylation [211,212] (Table 2).

#### Oxidative Stress in ADOA

Multiple mutations in *OPA1* have been identified in several ethnic groups, most of which are located at the GTPase domain and dynamin central regions [214], leading to different presentations of the disease. In this matter, a novel deletion of the GTPase domain of OPA1 was shown to alter mitochondrial morphology and distribution in fibroblasts obtained from three female individuals affected by ADOA in the same family [149]. However, the mitochondrial membrane potential, ATP synthesis rate, and RONS production were shown to be unaffected compared to controls, possibly indicating a less severe variant of the disease [149]. In addition, the expression of various OPA1 isoforms has been associated with increased susceptibility to oxidative stress and apoptotic cell death when measured in peripheral blood lymphocytes from ADOA patients incubated with 2-deoxy ribose [138].

Different degrees of genetic alterations may therefore correspond to clinical severity. Although ADOA typically presents during adolescence with insidious vision loss due to atrophy of the RGC layer with secondary loss of the optic nerve axonal fibers, a third of patients also show other symptoms such as sensorineural deafness, ataxia, ophthalmoplegia, and sensorimotor axonal polyneuropathy, as well as histological hallmarks of mitochondrial myopathy [215,216,217]. These individuals are denoted as ADOA plus. A recent study of four patient fibroblast lines harboring different OPA1 mutations in the GTPase or the C-terminal coiled-coil (ADOA plus) domains revealed mitochondrial elongation and complex IV defects in three of the four lines, which, however, were not associated with significantly lower oxygen consumption or ATP production [218]. Although fibroblasts carrying heterozygous *OPA1* mutations share significant mitochondrial remodeling with RGCs and thus may be useful for analysis of ADOA disease pathophysiology, RGCs may be more sensible to bioenergetic alterations given their higher metabolic demands [219,220,221]. In this regard, studies have also shown that mitochondrial calcium uptake and apoptosis in fibroblasts from ADOA patients correlate with symptom severity [139]. This can be interpreted in the sense that the more RGC and vision loss is present, the more the effects of ADOA will be represented in the fibroblasts. In this context, an additional study using ADOA plus patient-derived fibroblasts found increased mitochondrial fragmentation and depletion of mtDNA [156]. However, control fibroblasts undergoing siRNA-based knockdown of *OPA1* had more severe fragmentation and loss of mtDNA [156], indicating that the mitochondrial consequences may vary depending on the mutations in *OPA1*. Moreover and similarly to LHON, recent angiographic studies have identified a reduced microvascular network in the retina of ADOA patients that was associated with reductions in RNFL thickness [222,223,224]. The time course of these vascular changes and their association to different *OPA1* mutations need further investigation.

A recent study has drawn attention to the underdiagnosis of optic neuropathy due to *OPA1* mutations. This study sequenced the 29 exons of *OPA1* in 105 HAN Chinese patients suspected of suffering from LHON and found nine *OPA1* mutations (six in exons and three in splicing sites) in eight patients [225]. The authors then combined their data with 193 reported Han Chinese patients with optic neuropathy and compared these to the available data of 4327 East Asians by the Exome Aggregation Consortium (ExAC) and found a significant enrichment of potentially pathogenic *OPA1* mutations [225]. Some of these mutations were associated with alterations in mitochondrial morphology and cellular RONS concentration [225]. These findings suggest that the prevalence of *OPA1* mutations and optic neuropathies may be higher than previously assumed. Whether these findings are consistent within the general world population, however, has not yet been determined.

## 6. Optic Neuropathies Related to Environmental Health

### 6.1. Oxidative Stress in Toxic Optic Neuropathy

Only limited literature exists on toxicity-induced optic nerve damage. However, a few single case reports and case series have shed light on the association between toxic optic neuropathy and oxidative stress in response to medication, alcohol abuse, and exposure to toxic agents (Table 2). In particular, the consumption of a high-protein diet in combination with sleep (melatonin) and anti-depressant (selective serotonin reuptake inhibitor) medication was shown to induce central scotomas, defined as blind spots in the visual field, and bilateral visual acuity loss [226]. Visual acuity was improved when the normal diet was resumed and melatonin was discontinued [226]. Diets rich in protein stress mitochondrial metabolism of single amino acids, which, combined with other stressors such as a potential imbalance in the melatonin/dopamine turnover, can exacerbate a toxic insult to the retina and optic nerve accompanied by an increase in RONS. 

Certain drugs are also known to cause a decline in the antioxidant defense mechanisms, which may correlate with the development of toxic optic neuropathies. This is the case of ethambutol, an antibiotic used to treat tuberculosis that leads to alterations in the visual field and visual acuity in 2–6% of patients, depending on the dose and treatment duration [227]. Patients suffering ethambutol-induced toxic optic neuropathy were found to have lower levels of circulating SOD and catalase, especially if they were diabetic, compared to controls [144]. Moreover, RONS production is also a byproduct of excessive, chronic alcohol consumption, which has recently been associated with thinning of the neuroretinal rim, prolonged latency period, and no wave formation measured by a visual evoked potential test in a case series of ten males with daily alcohol consumption of 300 mL for at least 10 years [228]. Finally, it is also known that RONS alone cause optic neuropathy, which was observed in the case of a 51-year-old jeweler who broke and inhaled a bottle of hydrogen peroxide within 15 min [229]. In summary, toxic-induced mitochondrial and cellular stress and their dysfunction may either lead to increased oxidative stress, which can directly damage the retina and optic nerve, or exceed the tissue’s ability to clear RONS due to an altered antioxidant defense. 

### 6.2. Oxidative Stress in Nutritional Optic Neuropathy

The deficit of certain nutrients is also known to cause optic neuropathies [6,230,231] (Table 2). In this regard, a study performed on prisoners in Papua New Guinea investigated blood levels of several vitamins (a-tocopherol, b-carotene, lutein, folate, homocysteine, holotranscobalamin II, riboflavin, selenium, thiamin, and vitamins A, B12, and C) and the likelihood of developing optic neuropathy [145]. Decreased blood folate concentrations were evident in subjects with optic neuropathy, indicating that vitamin B9 deficiency may instigate the disease onset as well as its progression. Similar to these findings, an epidemiological and clinical study performed in Cuba found that the risk of developing optic neuropathy decreases with higher dietary intake of other B vitamins such as thiamine (B12), riboflavin (B2), and niacin (B3) [146]. Furthermore, higher serum concentrations of lycopene, an antioxidant carotenoid, were equally linked to a decreased risk of acquiring the disease [146]. Thus, further research on supplements to prevent vision loss associated with optic neuropathies may be worth considering.

## 7. Optic Disc Drusen

Optic disc drusen (ODD) are acellular deposits located in the prelaminar region of the optic nerve head and consist primarily of calcium, amino acids, nucleic acids, mucopolysaccharides, and a small amount of iron [232,233,234]. They are usually small but increase slowly in size and localize more superficially over time. ODD are found in approximately 2% of the population and are primarily a bilateral phenomenon [235]. ODD are associated with visual field defects, which are present in up to 87% of the cases, and with peripapillary retinal nerve fiber layer damage [236,237,238]. ODD can also cause sudden visual loss due to vascular complications such as retinal vascular occlusion and hemorrhage, subretinal neovascularization, and anterior ION [239]. 

The pathogenesis of ODD has yet to be understood. However, several mechanisms have been proposed [235]. According to histochemical studies, ODD originate from axoplasmic derivatives and are a by-product of abnormal axonal metabolism [233,240,241]. Using electron microscopy, Tso et al. observed needle-like deposits suggesting calcium accumulation in the mitochondria, both in the intracellular milieu and among dilated and ruptured axons [241]. Such deposits display a variable extent of calcification, the highest being in the extracellular mitochondria. Hence, it was hypothesized that some axons rupture, resulting in the release of mitochondria to the extracellular space, where they calcify into small microbodies. These microbodies fuse over time due to continuous accumulation of axoplasm, which is further calcified forming ODD [241]. In addition, intra-axonal material may also be extruded due to swelling and congestion of the prelaminar nerve fibers secondary to a small scleral canal, which is characteristic of patients with ODD [239,242,243]. 

### Oxidative Stress in Optic Disc Drusen

Mitochondrial calcification is a cellular mechanism to protect cells from elevated calcium concentrations that can lead to cytotoxicity [244]. Oxidative stress stimulates mitochondrial calcium overload by promoting its influx and, at the same time, calcium is known to promote the generation of RONS by the mitochondria [245]. Inflammation and hypoxia also lead to the accumulation of ions in the mitochondria initiating the process of intramitochondrial mineral formation, including calcification [244,246]. In this regard, hypoxia may play a significant role in ODD pathogenesis since reduced retinal vascular density and retinal blood flow have been observed in patients with ODD together with a positive correlation between vascular density and RNFL thickness [247,248,249,250,251,252]. Since intracellular calcification is primarily mediated by the mitochondria, these organelles are crucial for the maintenance of cellular calcium homeostasis [244]. Given the chronic nature of ODD and the congestion of the prelaminar nerve fibers, one might hypothesize that calcium homeostasis can be affected in these nerve fibers secondary to an oxidative stress phenomenon associated, or not, with vascular changes. However, this hypothesis has not been investigated yet.

## 8. Conclusions and Future Perspectives

Redox imbalance and oxidative stress appear to be consistent among optic neuropathies, especially in POAG, PEXG, LHON, and ADOA given the higher amount of information gathered about these diseases over the last years and could certainly have an important role in the pathogenesis that leads to RGC neurodegeneration. In particular, increased levels of oxidative stress markers and changes in antioxidant defense mechanisms that affect both antioxidant enzymes and glutathione, the main antioxidant molecule present in the organism, are noticeable. However, some study limitations make it difficult to extract solid conclusions and establish the causal events leading to oxidative stress in these patients. The first and most obvious is patient stratification, especially in glaucoma. The complex nature of this multifactorial disease, which includes different glaucoma subtypes, is usually ignored when assessing changes in oxidative stress and only a few recent studies characterized and divided glaucoma patients into subgroups for data analysis (e.g., NTG and HTG patients). Furthermore, the population size in some studies was certainly small, especially in the optic neuropathies that are less common among the population such as NAION or optic neuritis, which decreases the statistical power of analysis. Another factor that may limit the scope of some studies and possibly lead to conflicting results is the inclusion criteria. For example, most of the studies cited in this review excluded patients with systemic diseases such as diabetes, while a few studies included patients with these conditions if they were controlled with medication. Oxidative stress-related parameters can certainly be affected by systemic disorders and/or off-target related effects of drug therapy, which may introduce a confounding factor that can mask a real association or falsely show an apparent association between study variables when this one is not real. Finally, although human studies are advantageous compared to animal experiments because they allow assessment of particular parameters and/or biomarkers directly in the patient, ethical considerations limit access to biological samples. For example, aqueous humor sampling can only be performed when patients are subjected to an ocular intervention to treat a specific pathology (i.e., cataracts), which limits the sample size and type of control group. Further research should aim at improving study design, sample size, and patient stratification to obtain robust information about redox dysregulations associated with optic neuropathies. 

## Figures and Tables

**Table 1:** Glaucoma.

Type of Glaucoma	Type of Sample	Outcome (Related to Control Group) *	Country	Authors
*General antioxidant/oxidant status*				
POAG	Blood	Lower TAC	Italy	[45]
POAG	Plasma	Lower TAS. Negative correlation with glaucoma severity	Saudi Arabia	[46]
POAG	Blood	Lower BAP. Negative correlation with visual acuity	Japan	[47]
POAG	Serum	Lower BAP. Positive correlation with RGCs density in young males (<65 years old)	Japan	[48]
PEXG	Plasma	Lower TAS	Saudi Arabia	[49,50]
PEXG	Serum	Lower TAC	Turkey	[51,52]
**POAG**	Aqueous humor	Lower TAC	Italy	[45]
**POAG**	Aqueous humor	Lower TAC	Spain	[53]
**POAG**	Aqueous humor	Lower TAC	Argentine	[54]
**PEXG**	Aqueous humor	Lower TAC	Turkey	[51]
**PEXG**	Aqueous humor	Lower TRAP	Argentine	[55]
**PEXG**	Aqueous humor	Higher TAS	Turkey	[56]
PEXG	Serum	Higher TOS	Turkey	[51,52]
**PEXG**	Aqueous humor	Higher TOS	Turkey	[51]
**PEXG**	Aqueous humor	No changes	Turkey	[56]
*Antioxidant defense mechanisms*				
POAG	Peripheral blood mononuclear cells	Higher levels of GSSC and lower GSH-to-GSSC ratio. Positive correlation between GSH-to-GSSC ratio and visual field damage	Japan	[57]
POAG (IOP ≥ 24 mm Hg)	Blood	Lower levels of GSH and total GSH	England	[58]
POAG (IOP ≥ 24 mm Hg) and NTG	Blood	Lower levels of GSH and total GSH in POAG and NTG. Lower redox index in POAG (IOP ≥ 24 mm Hg)	England	[59]
PEXG	Plasma	Higher GSH levels	Turkey	[60]
POAG	Serum	Higher disulfide, disulfide-to-native thiol ratio, disulfide-to-total thiol ratio	Turkey	[61]
POAG	Blood	Downregulation of SOD1 mRNA and upregulation of SOD2 mRNA Upregulation of GPX1 mRNA	Colombia	[62]
POAG	Serum	Lower activity of SOD2 but no changes in SOD1 activity	Poland	[63]
PEXG	Plasma	Higher total SOD activity	Turkey	[52,64]
PEXG	Blood	Lower total SOD activity Lower catalase activity	Turkey	[60]
PEXG	Serum	Lower catalase activity	Greece	[65]
**POAG**	Aqueous humor	Increased total SOD activity Increased total GPX activity No changes in catalase activity	Argentine	[54]
**POAG**	Aqueous humor	Increased total SOD activity Increased total GPX activity No changes in catalase activity	Egypt	[66]
**POAG and PACG**	Aqueous humor	Increased total SOD and GPX activity in both POAG and PACG No changes in catalase activity	India	[67]
**POAG**	Aqueous humor	Increased total SOD activity	Spain	[53]
**PEXG**	Aqueous humor	Higher total SOD activity	Argentine	[55]
**PEXG**	Aqueous humor	Lower catalase activity	Greece	[65]
**PEXG**	Aqueous humor	Higher total GPX activity	Argentine	[55]
*Reactive oxygen and nitrogen species (RONS)*				
POAG	Serum	Higher ferritin levels, especially in men	South Korea	[68,69]
PEXG	Plasma	Higher NO levels	Turkey	[52]
PEXG	Blood	Lower NO levels	Turkey	[60]
**POAG and PACG**	Aqueous humor	Increased NO levels	Egypt	[66]
**POAG and PACG**	Aqueous humor	Increased NO levels	Taiwan	[70]
**POAG**	Trabecular meshwork	Upregulation of iNOS expression and activity and downregulation of calcium-dependent NOS expression and activity. Positive correlation with visual field defects	Spain	[71]
*Oxidative stress markers*				
POAG	Plasma	Higher 8-OHdG levels	Saudi Arabia	[72]
POAG	Plasma	Higher 8-OHdG levels and lower PARP1 and OGG1 levels. Negative correlation between PARP1 and OGG1 expression and 8-OHdG levels	India	[73]
POAG	Blood	Higher 8-OHdG levels	Turkey	[74]
PEXG	Plasma	Higher 8-OHdG	Saudi Arabia	[75]
**POAG**	Aqueous humor	Higher 8-OHdG levels	India	[73]
**POAG**	Trabecular meshwork	Higher 8-OHdG levels. Positive correlation with visual field defects	Italy	[76,77]
POAG	Blood	Higher MDA levels	Turkey	[74]
POAG	Blood	Higher MDA levels	Spain	[45]
POAG	Serum	Higher MDA levels	Poland	[63]
NTG and HTG	Serum	Oxidation products of linoleic and arachidonic acid increased in both NTG and HTG	Japan	[78]
PEXG	Plasma	Higher MDA levels	Turkey	[52]
PEXG	Blood	Higher MDA levels	Turkey	[60]
**POAG**	Aqueous humor	Higher MDA levels	Italy	[45]
**POAG**	Aqueous humor	Higher MDA levels	Spain	[79]
**POAG (excluded NTG)**	Aqueous humor	No changes in MDA levels	Spain	[71]
**POAG (excluded NTG)**	Trabecular meshwork	Higher nitrotyrosine immunoreactivity. Positive correlation with higher IOP	Spain	[71]
**POAG**	Blood vessels and glia in the ONH	Higher nitrotyrosine immunoreactivity	Canada	[80]

Abbreviations: BAP: biological antioxidant potential, GPX: glutathione peroxidase, GSH: glutathione, GSSG: oxidized glutathione, HTG: high tension glaucoma, MDA: malondialdehyde, NO: nitric oxide, NOS: NO synthase, NTG: normal-tension glaucoma, OGG1: oxoguanine DNA glycosylase, PARP1: poly (ADP-ribose) polymerase, PEXG: pseudoexfoliation glaucoma, POAG: primary open-angle glaucoma, TAC: total antioxidant capacity, TAS: total antioxidant status, TOS: total oxidant status, TRAP: total reactive antioxidant potential, SOD: superoxide dismutase, 8-OHdG: 8′-hydroxy-2′-deoxyguanosine. * Control group: may refer to patients with other eye diseases not associated with optic nerve degeneration (e.g., cataracts), healthy unrelated subjects (e.g., spouses), healthy relatives (e.g., mutation carriers not suffering the optic neuropathy), etc. Results obtained from systemic samples (e.g., plasma) are written in black and results obtained from ocular samples (e.g., aqueous humor) are depicted in grey.

## Abbreviations

8-OHdG: 8′-hydroxy-2′-deoxyguanosine, ACG: angle-closure glaucoma, ADOA: autosomal dominant optic atrophy, AION: arteritic ischemic optic neuropathy, AOPP: advanced oxidation protein products, ATP: adenosine triphosphate, BAP: biological antioxidant potential, BER: base excision repair, CI-V: protein complexes I-V, CNS: central nervous system, CSC: cigarette smoke condensate, Cu: copper, ExAC: Exome Aggregation Consortium, GGT: gamma glutamyltransferase, GPX: glutathione peroxidase, GSH: reduced glutathione, GSSG: oxidized glutathione, *GSTM1*: isoform of glutathione s-transferase Mu 1, *GSTT1*: isoform of glutathione s-transferase Theta 1, hiPSCs: human induced pluripotent stem cells, HTG: high-tension glaucoma, ION: ischemic optic neuropathy, IOP: intraocular pressure, LHON: Leber’s hereditary optic neuropathy, MDA: malondialdehyde, Mg: manganese, MS: multiple sclerosis, mtDNA: mitochondrial DNA, NAION: non-arteritic ischemic optic neuropathy, NMO: neuromyelitis optica, NO: nitric oxide, eNOS: endothelial nitric oxide synthase, iNOS: inducible nitric oxide synthase, nNOS: neuronal nitric oxide synthase, NTG: normal-tension glaucoma, OAG: open-angle glaucoma, ODD: optic disc drusen, OGG1: oxoguanine DNA glycosylase 1, OHT: ocular hypertension, ONH: optic nerve head, OSI: oxidative stress index, PACG: primary angle-closure glaucoma, PARP1: poly (ADP-ribose) polymerase, PEXG: pseudoexfoliation glaucoma, POAG: primary open-angle glaucoma, RGC: Retinal ganglion cell, RNFL: retinal nerve fiber layer, RONS: reactive oxygen and nitrogen species, SOD: superoxide dismutase, TAC: total antioxidant capacity, TAS: total antioxidant status, TOS: total oxidant status, TRAP: total reactive antioxidant potential, XRCC1: X-ray repair cross-complementing 1, Zn: zinc.
